# Papillary Thyroid Carcinoma With Laryngotracheal Intraluminal Invasion Requiring Total Laryngectomy: A Case Report From Nepal

**DOI:** 10.7759/cureus.113986

**Published:** 2026-08-05

**Authors:** Subhash Devkota, Dinanka Subedi, Kamana Chalise, Prabhat Sharma

**Affiliations:** 1 Otolaryngology - Head and Neck Surgery, Chitwan Medical College, Chitwan, NPL; 2 Otolaryngology, B.P. Koirala Memorial Cancer Hospital, Chitwan, NPL

**Keywords:** airway obstruction, laryngectomy, papillary, thyroid cancer, thyroidectomy, tumor invasiveness

## Abstract

Papillary thyroid carcinoma (PTC) is the most common thyroid malignancy and usually carries a favorable prognosis. However, invasion of adjacent aerodigestive structures such as the larynx and trachea is rare and is associated with increased morbidity and mortality. A 53-year-old female presented with a progressively enlarging anterior neck swelling, intermittent hemoptysis, and progressive dyspnea. Imaging and histopathology confirmed PTC with invasion into the trachea and larynx. The patient underwent total laryngectomy, total thyroidectomy, bilateral modified radical neck dissection, and central compartment neck dissection. Histopathology confirmed the follicular variant of PTC with extrathyroidal extension and nodal metastasis. In cases of PTC with suspected tracheal or laryngeal invasion, timely imaging and aggressive surgical management are crucial for optimizing outcomes.

## Introduction

Papillary thyroid carcinoma (PTC) is the most common thyroid malignancy, accounting for approximately 85% of thyroid cancers, with a female-to-male ratio of approximately 3:1 and a peak incidence between the third and fifth decades of life. PTC generally has an excellent prognosis due to its indolent course and responsiveness to therapy [1]. Tumors typically spread via lymphatics but rarely invade adjacent aerodigestive structures such as the larynx, trachea, or esophagus in 0.5% to 1.5% of cases [2].

Direct laryngotracheal invasion can cause morbidities ranging from hoarseness, aspiration, and hemoptysis to life-threatening airway obstruction [3]. Depending on the depth and extent of invasion, the management protocol varies from conservative tumor shaving procedures to circumferential laryngeal resection to achieve a clear oncological margin [4].

As most differentiated thyroid carcinomas are diagnosed before extensive intraluminal destruction occurs, radical resections such as total laryngectomy are required in only 1% to 3.5% of cases [5]. Therefore, the opportunity to observe the natural progression of untreated intraluminal disease is rare. We report this case to document the natural progression of locally advanced PTC and to highlight a key diagnostic pitfall: normal nasopharyngoscopy findings do not exclude subglottic or upper tracheal mucosal invasion; therefore, lower airway endoscopy and imaging are required whenever aerodigestive invasion is clinically suspected.

This article was previously posted as a preprint on the Authorea preprint server on February 9, 2026 (DOI:10.22541/au.177063332.25413078/v1).

## Case presentation

A 53-year-old female presented to the outpatient department in April 2024 with a five-month history of anterior neck swelling, two months of intermittent hemoptysis, and one month of progressive dyspnea. The neck swelling was painless, progressive, and not associated with hoarseness, dysphagia, or weight loss. Hemoptysis was scanty, with fresh blood mixed in sputum occurring once to twice monthly. Dyspnea had gradually worsened from occurring on exertion to during minimal activity.

On examination, there was a 2 × 2 cm anterior neck nodule, mobile on deglutition but not on tongue protrusion. No skin changes, venous engorgement, or lymphadenopathy were noted. The swelling was firm, non-tender, and fixed to deeper structures. Laryngeal crepitus was present, and the trachea was central. No cervical lymph nodes were palpable.

**Figure 1:**
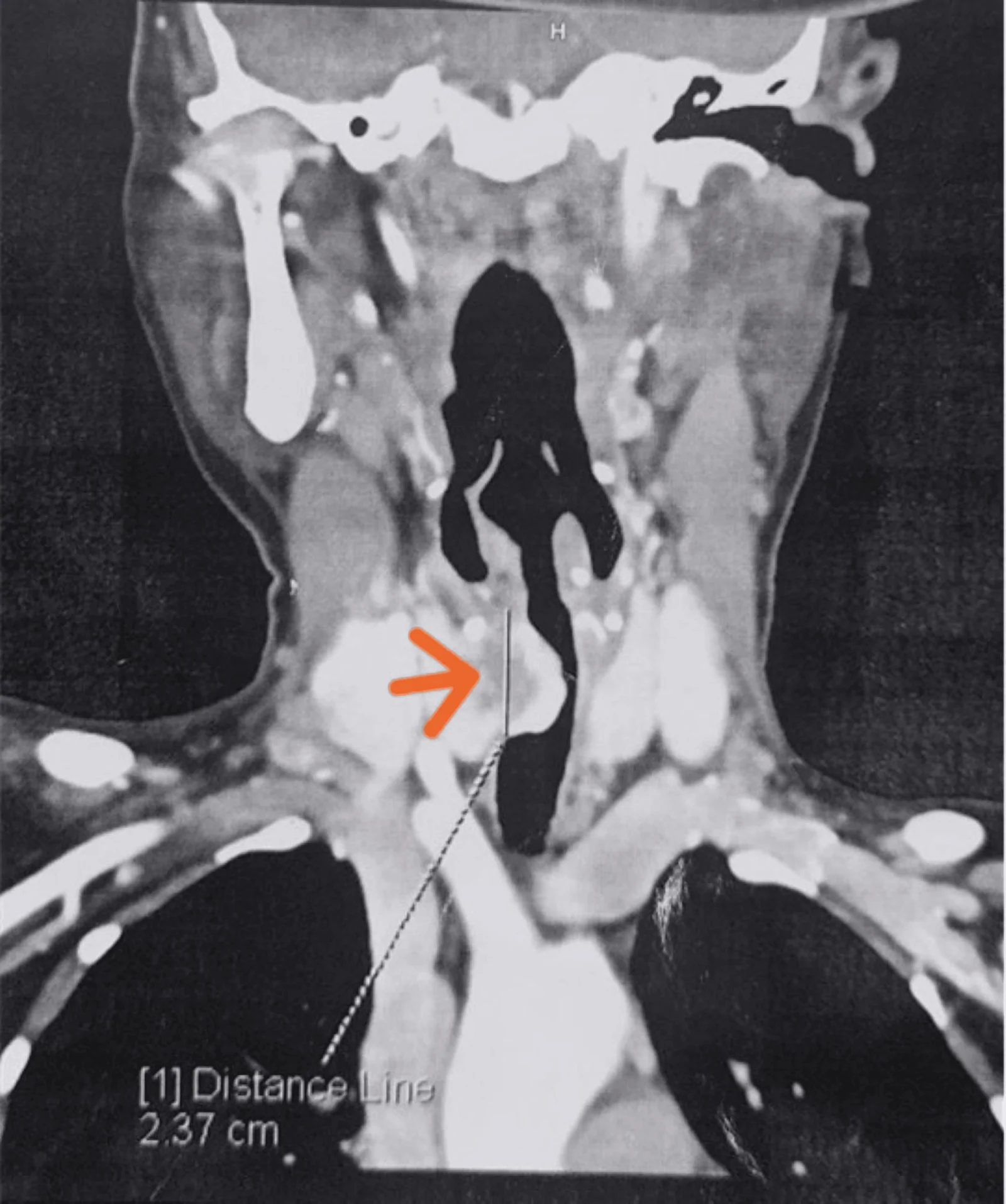
Contrast-enhanced computed tomography of the neck (coronal view) demonstrating a tumor infiltrating the larynx and trachea (orange arrow).

Contrast-enhanced CT of the neck and chest revealed a hypodense, enhancing mass (2.5 × 3.2 × 2.6 cm³) in the mid to superior pole of the right thyroid lobe and isthmus, with multiple calcifications. The lesion invaded the strap muscles and right cricothyroid joint and projected into the tracheal lumen, causing narrowing from the subglottis to the third tracheal ring (Figure 1). Enlarged, calcified lymph nodes were noted in right levels III and IV.

**Figure 2:**
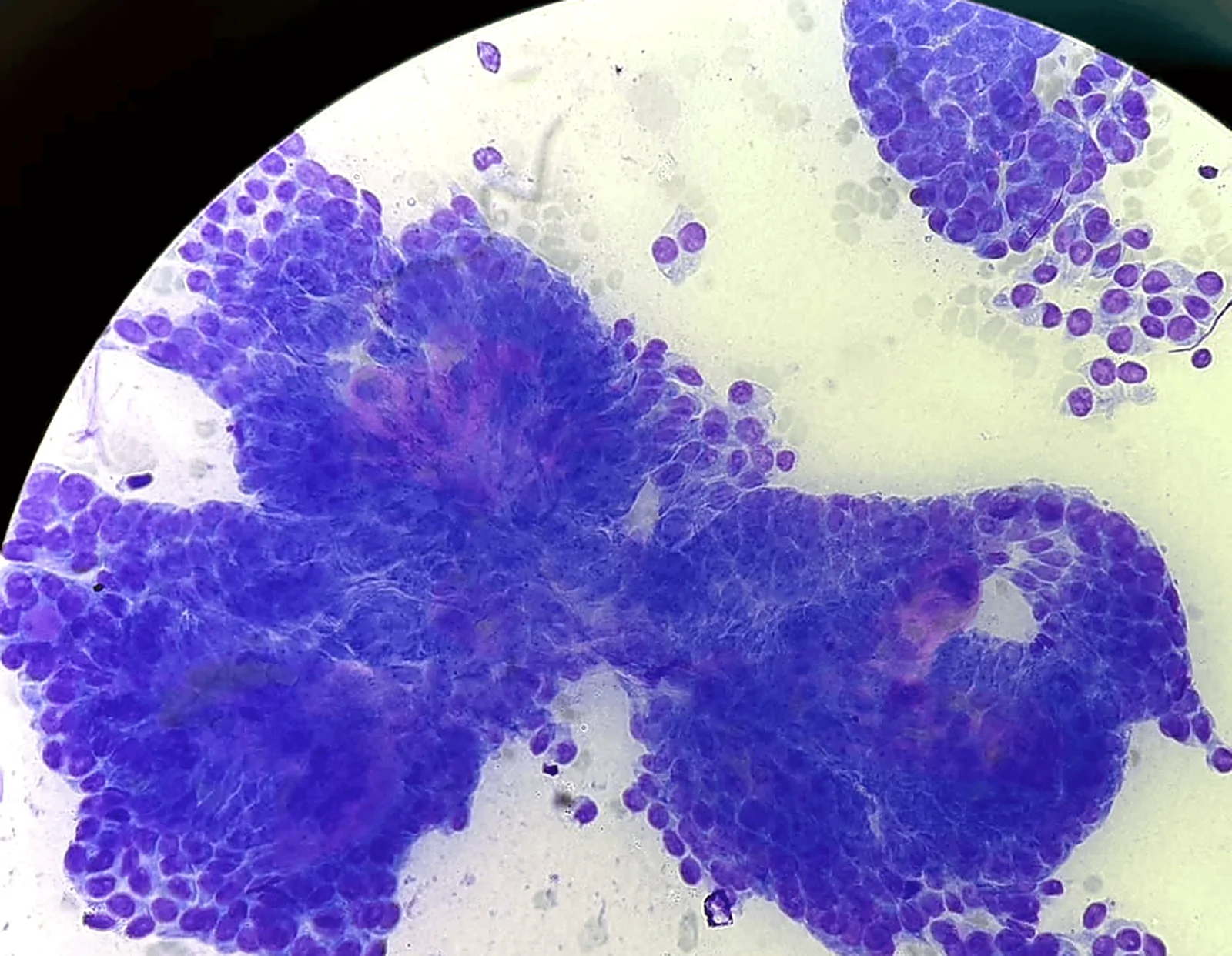
Fine-needle aspiration cytology showing branching papillary fragments consistent with papillary thyroid carcinoma (hematoxylin and eosin, 40× magnification).

Fine-needle aspiration cytology (FNAC) was suggestive of PTC (Figure 2).

**Figure 3:**
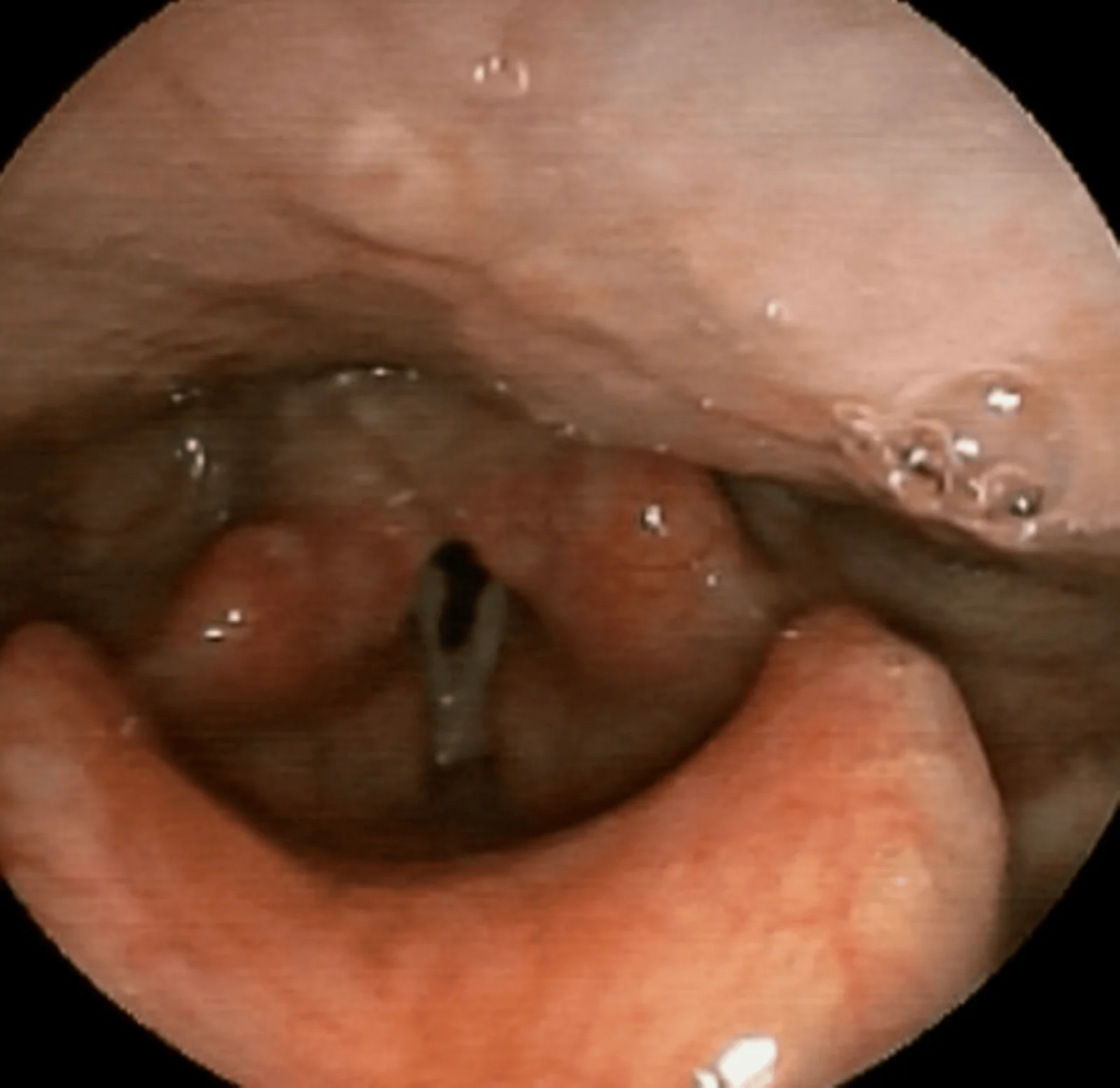
Flexible nasopharyngolaryngoscopy showing a normal-appearing endolarynx with a patent glottis and unremarkable mucosa.

Nasopharyngolaryngoscopy showed a normal endolarynx with preserved vocal cord mobility (Figure 3).

**Figure 4:**
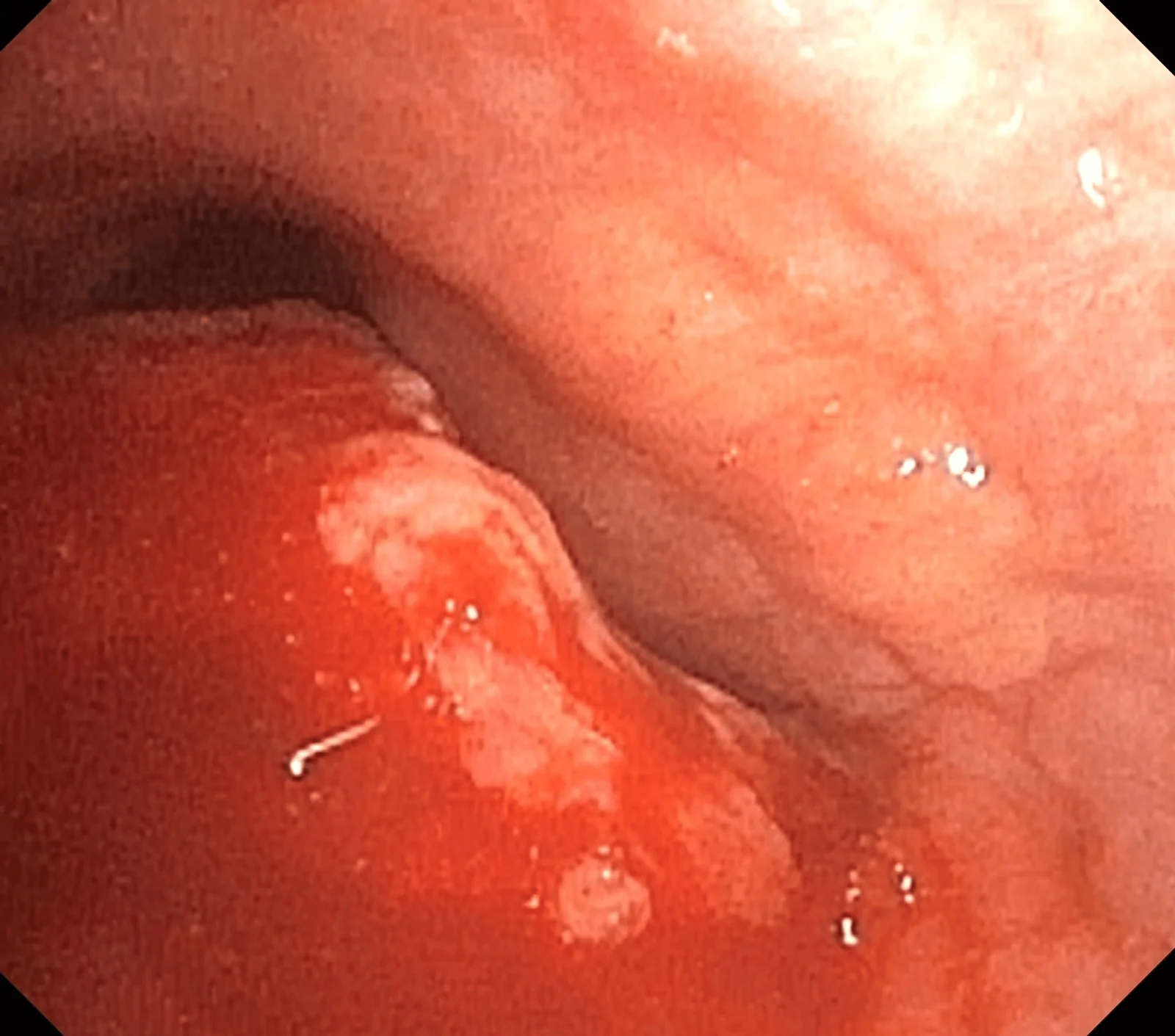
Bronchoscopic view showing an ulceroproliferative lesion extending from the subglottis to the trachea causing luminal compromise. The lesion appears erythematous, irregular, and friable with contact bleeding.

Bronchoscopy revealed an ulceroproliferative lesion extending approximately 1 cm below true vocal cords to the level of the third tracheal ring, causing luminal compromise. The lesion appeared erythematous, irregular, and friable with contact bleeding, causing approximately 50%-60% asymmetric cross-sectional narrowing of the airway. Biopsy was not performed considering the risk of bleeding (Figure 4).

Upper gastrointestinal endoscopy was normal. PTC with extrathyroidal extension was considered the most likely diagnosis due to the indolent clinical course, presence of a calcified thyroid-based mass on imaging, and FNAC findings consistent with PTC. Anaplastic carcinoma was considered but was less likely given the absence of rapid progression, constitutional symptoms, or vocal cord paralysis. Thyroid lymphoma was also considered but was unlikely due to the focal nature of the lesion, absence of Hashimoto’s thyroiditis, and presence of calcifications. Primary laryngeal malignancy was excluded as the lesion originated from the thyroid gland with secondary laryngeal invasion and normal endolaryngeal findings. The lesion infiltrated the laryngeal and tracheal cartilage to involve the mucosa with intraluminal extension, consistent with Shin stage IV laryngotracheal invasion [6].

**Figure 5:**
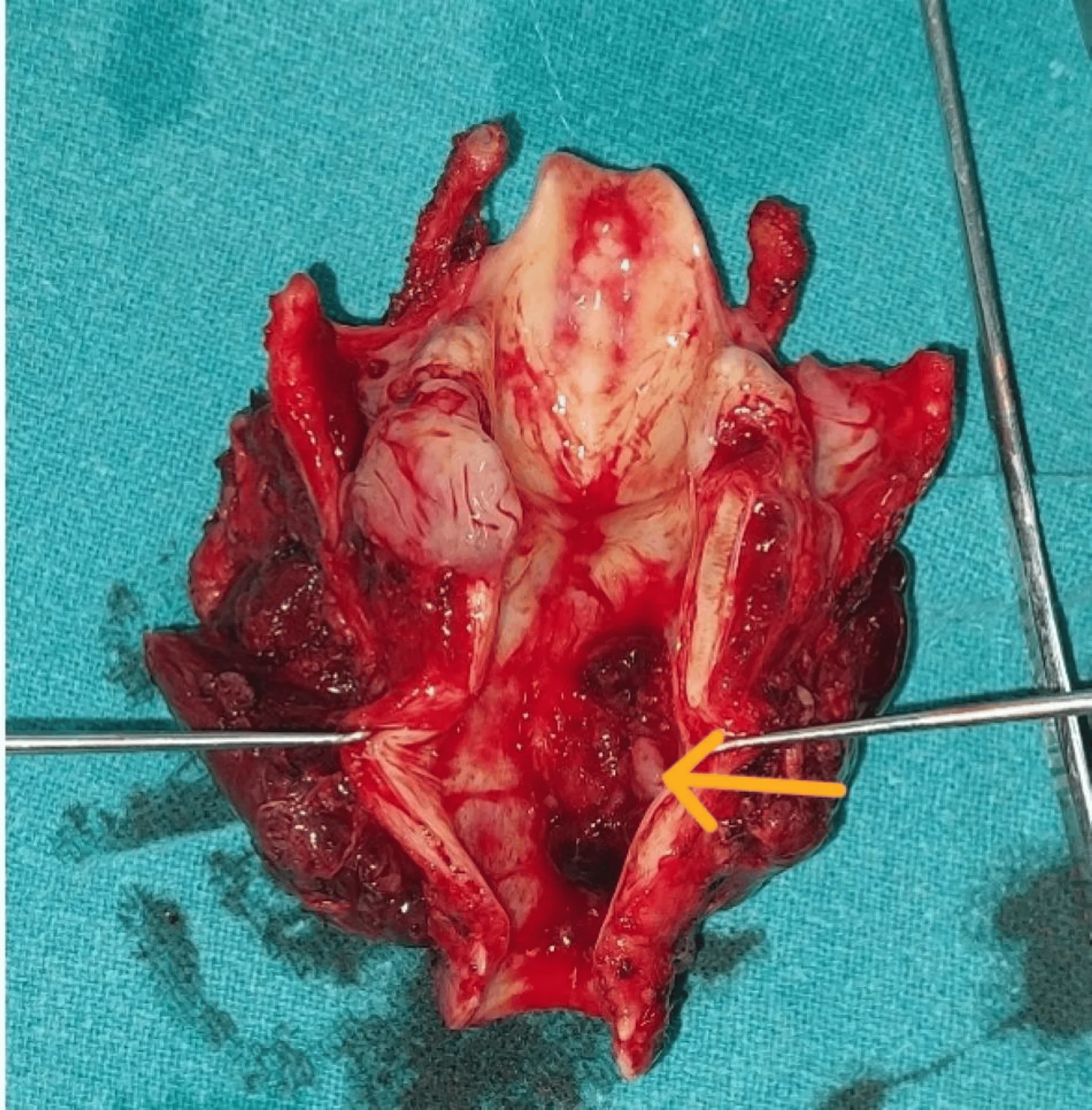
Resected laryngotracheal specimen demonstrating an ulceroproliferative lesion (yellow arrow).

The patient underwent total laryngectomy, total thyroidectomy, bilateral modified radical (Type III) neck dissection [7], and central compartment (Level VI) neck dissection. Intraoperatively, a 2 × 2 cm mass in the right thyroid lobe infiltrated the tracheal cartilage, extending from the subglottis up to the tracheal lumen. Significant nodal involvement was noted at right levels IIa, III, IV, and bilateral level VI. The resected laryngotracheal specimen demonstrated a gross ulceroproliferative lesion (Figure 5).

**Figure 6:**
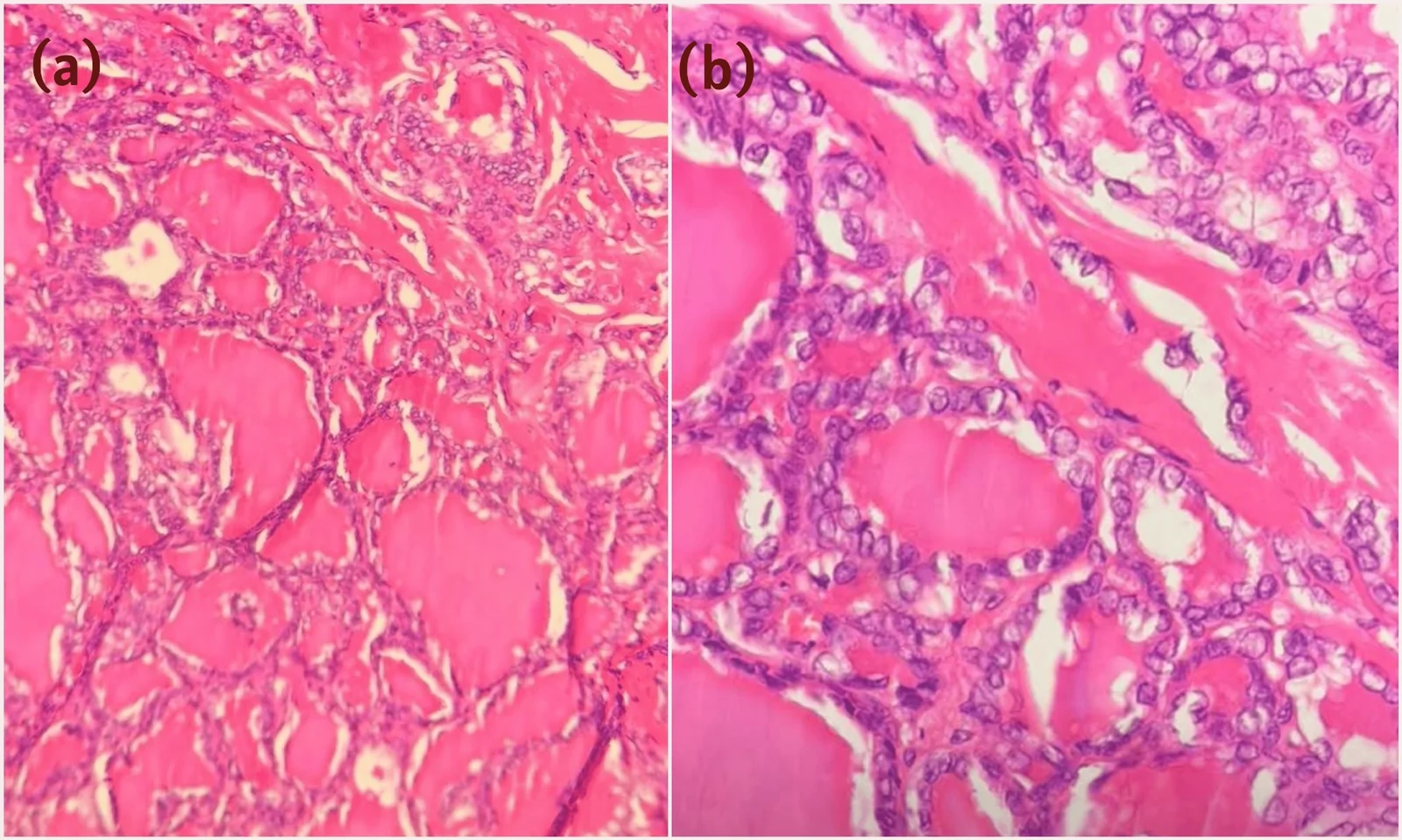
Available photomicrographs of the histopathological features of the thyroid tumor with laryngotracheal invasion (hematoxylin and eosin). (a) Low-power view showing a follicular architectural pattern with follicles of variable size separated by thin fibrovascular septae. (b) High-power view showing variably sized follicles lined by follicular epithelial cells demonstrating nuclear features of papillary thyroid carcinoma. The follicular lumen contains dense eosinophilic colloid. Note: The reproduced photomicrographs are limited by resolution; the diagnosis was based on direct microscopic examination of the original slides, together with clinicopathological correlation, rather than on the reproduced images alone.

Histopathology revealed the follicular variant of PTC involving the right thyroid lobe, measuring 2.6 × 2.3 × 2.3 cm, and unifocal in distribution (Figure 6). The tumor demonstrated capsular breach with gross extrathyroidal extension into the strap muscles, adjacent soft tissue, larynx, and trachea. Lymphovascular and perineural invasion were both identified. All surgical resection margins were uninvolved by invasive carcinoma. Of the 43 cervical lymph nodes examined from the bilateral neck dissection specimen, six were positive for metastatic PTC (three at level VI, three at right level IV), with extranodal extension present in one of the three metastatic level IV nodes. The tumor was finally staged as pT4a pN1b pM0. The postoperative course was uneventful.

The patient completed radioactive iodine ablation with 150 mCi of I-131 12 weeks post-surgery. A radioiodine whole-body scan performed six months after ablation showed no evidence of disease. There was no relapse till 18 months after surgery. The patient had mild swallowing difficulty, particularly with solid foods, resulting in prolonged meal time. Voice rehabilitation was achieved through esophageal speech, with limited voice quality. The patient is under regular follow-up with a speech-language pathologist for voice rehabilitation.

## Discussion

PTC is typically indolent but may exhibit aggressive behavior in certain variants. Poor prognostic factors include patient age over 55 years, aggressive histological subtypes (e.g., tall cell, diffuse sclerosing), extrathyroidal extension, and distant metastasis [8].

Extrathyroidal extension occurs in approximately 5%-34% of cases and is a key factor in determining prognosis and staging [9]. Invasion into the upper aerodigestive tract structures such as the larynx or trachea is rare (0.5%-1.5%) and may be encountered in patients with delayed presentation or diagnosis [4]. Intraluminal invasion carries a worse prognosis than mere cartilage invasion, with airway obstruction being a major cause of mortality. Although nasopharyngolaryngoscopy showed a normal endolarynx with preserved vocal cord mobility in this patient, bronchoscopy revealed an ulceroproliferative lesion extending from the subglottis to the level of the third tracheal ring, causing luminal compromise. This highlights that conventional laryngoscopy may miss subglottic or tracheal extension. Therefore, when there is strong clinical suspicion, comprehensive evaluation with cross-sectional imaging and lower airway endoscopic assessment should be considered.

Despite aggressive local invasion, complete surgical excision supplemented by adjuvant radioiodine therapy or radiotherapy can lead to favorable outcomes [10]. Unlike squamous cell carcinomas, thyroid carcinomas may be resected with close margins as well and treated further with I-131 or external beam radiotherapy. Therefore, their resection should be approached differently [3].

The optimal surgical approach in cases of laryngeal invasion remains controversial. Options range from conservative “shaving” to radical resections such as total laryngectomy. In cases of intraluminal extension or hemorrhage, radical excision is often necessary for airway protection and complete tumor removal [4]. According to the classification proposed by Brauckhoff et al., total laryngectomy is indicated in thyroid carcinoma with bilateral laryngeal invasion or extensive vertical tracheal invasion [11]. Total laryngectomy was performed in this patient because of the extensive subglottic mucosal involvement extending up to the third tracheal ring, where complete oncologic resection with adequate margins and preservation of a functional larynx were difficult. The total laryngectomy procedure helped to achieve negative surgical margins and satisfactory locoregional disease control.

According to the literature, total laryngectomy is performed in only 1%-3.5% of differentiated thyroid carcinomas, as many patients undergo treatment before disease progresses to this extent. Opportunity to observe the natural course of PTC is rare. This case illustrates the natural progression of untreated PTC [5].

According to the 2025 American Thyroid Association Guideline for Differentiated Thyroid Cancer [12], gross extrathyroidal extension beyond the capsule is considered T4. According to the radiological and histopathological findings of this case, TNM staging was T4aN1bM0, which has a 10-year survival rate of 75.9% [13]. Management goals of such advanced cases include curative resection, palliation, and improvement in quality of life and survival. A multidisciplinary approach is necessary for management of such cases.

A unique feature of this case is unusual rapid clinical progression. The patient presented with only five months of symptoms, but the tumor had already invaded the larynx and trachea, with intraluminal extension causing airway compromise. PTC generally spreads slowly and has an excellent prognosis. Histopathological diagnosis of the follicular variant of PTC exhibiting this aggressive pattern of spread is uncommon. These features make this case a valuable addition to the limited literature.

## Conclusions

Laryngotracheal invasion by PTC is uncommon and reflects advanced local disease. This case demonstrates that complete tumor resection may require radical procedures, such as total laryngectomy, in selected patients with Shin stage IV disease to achieve a negative oncologic margin. Furthermore, normal endolarynx with preserved vocal cord mobility on nasopharyngolaryngoscopy does not exclude subglottic or tracheal mucosal involvement, highlighting the complementary role of bronchoscopy and cross-sectional imaging when aerodigestive extension is suspected.
